# Predictive Value of Mean Platelet Volume in Variceal Bleeding due to Cirrhotic Portal Hypertension

**DOI:** 10.5005/jp-journals-10018-1203

**Published:** 2017-05-05

**Authors:** Mehmet A Erdogan, Ali R Benli, Serap B Acmali, Mustafa Koroglu, Yahya Atayan, Ahmet Danalioglu, Burcak Kayhan

**Affiliations:** 1 Department of Gastroenterology, Karabük Education and Research Hospital, Karabük University, Karabük, Turkey; 2 Department of Family Medicine, Karabük University, Karabük, Turkey; 3 Department of Family Medicine, Dudullu Aile Sağliği Merkezleri, Istanbul Turkey; 4 Department of Hematology, Karabük University, Karabük, Turkey; 5 Department of Gastroenterology, Gümüshane State Hospital Gümüşhane, Turkey; 6 Department of Internal Medicine, Bezmiâlem Vakif University, Istanbul, Turkey; 7 Department of Internal Medicine Karabük University, Karabük, Turkey

**Keywords:** Cirrhosis, Mean platelet volume, Portal hypertension, Variceal bleeding.

## Abstract

**Aim:**

To investigate whether mean platelet volume (MPV) is a predictor of variceal bleeding in patients with cirrhotic portal hypertension.

**Materials and methods:**

This prospective cohort was performed in the internal medicine department of our tertiary care center. Cirrhotic patients were allocated into two groups: Group I consisted of 31 cases without a history of variceal bleeding, whereas group II was made up of 31 patients with a history of variceal bleeding. Data derived from medical history, physical examination, ultrasonography, gastrointestinal system endoscopy, complete blood count, hepatic, and renal function tests were recorded and compared between two groups. On physical examination, encephalopathy and ascites were evaluated and graded with respect to Child-Pugh-Turcotte classification.

**Results:**

There was no significant difference between the two groups in terms of age, duration of the disease, and gender of the patient. The only remarkable difference was that hemoglobin (p = 0.02) and hematocrit (p = 0.02) values were lower in group II. Neither the etiology of bleeding was different between groups nor did MPV seem to have a noteworthy impact on bleeding. Interestingly, risk of variceal bleeding increased in parallel to the higher grade of varices.

**Conclusion:**

Our results imply that there is a correlation between the grade of varices and esophageal vari-ceal bleeding in cirrhotic patients. However, association between MPV and variceal bleeding could not be demonstrated. Utilization of noninvasive tests as predictors in these patients necessitates further controlled trials on larger series.

**How to cite this article:** Erdogan MA, Benli AR, Acmali SB, Koroglu M, Atayan Y, Danalioglu A, Kayhan B. Predictive Value of Mean Platelet Volume in Variceal Bleeding due to Cirrhotic Portal Hypertension. Euroasian J Hepato-Gastroenterol 2017;7(1):6-10.

## INTRODUCTION

One of the most important causes of mortality in patients with cirrhosis and portal hypertension is upper gastrointestinal system (GIS) bleeding. In routine clinical practice, assessment of the risk of upper digestive tract bleeding relies on esophagogastroduodenoscopy (EGD). Even though prediction of the occurrence of bleeding attributed to esophageal varices in cirrhosis is mostly difficult, clinical and endoscopic signs that point out a higher risk of bleeding have been reported. Some of these clues include the size of the esophageal varices, the presence of cherry-red spots, and the severity of cirrhosis evaluated according to the Child-Pugh classification.^1^ Since esophageal variceal bleeding comprises 80 to 90% of all bleeding from the upper GIS in cirrhotic patients, timely recognition and classification of patients under a high risk of variceal bleeding is critical.^2^

The gold standard for assessment of risk of vari-ceal bleeding is EGD, possibly with endoscopic color Doppler ultrasonography.^3^ However, despite its advantages, EGD is an unpleasant and expensive test for regular follow-up and also carries the risk of bleeding due to manipulation, especially in patients with large varices. Cirrhotic patients frequently undergo screening endoscopy for the diagnosis or follow-up of esophageal varices. However, in the future, this social and medical burden will increase due to the greater number of patients with chronic liver disease and their prolonged survival.^4^ There is evidence for utilization of alternative methods including ultrasonography rather than endoscopy for diagnosis of esophageal varices.^5^

Platelet indicators and, particularly, mean platelet volume (MPV) are supposed to demonstrate the platelet activity by means of platelet swelling and pseudopodia formation.^6,7^ In other words, the greater platelet size means increased numbers of platelet are activated. Accordingly, it can be suggested that larger platelets are associated with a more reactive etiology and increased risk for thromboembolism.^8,9^ Furthermore, Chu et al^10^ have shown that MPV values were higher in patients with acute myocardial infarction and these values were associated positively with an increased rates of mortality. Patients with low MPV may be vulnerable for bleeding complications and this link has been already reported by Magri et al.^11^ In contrast, Huczek et al^6^ found that high values of MPV may be associated with increased risk of bleeding complications after trans-catheter aortic valve implantation. Also, Moghaddam et al^12^ noted higher MPV levels in a group of preterm infants with intraventricular hemorrhage and broncho-pulmonary dysplasia. Therefore, identification of novel clinical, biochemical, and ultrasonographic parameters that may noninvasively and more practically predict the presence of esophageal variceal bleeding in cirrhosis is an important issue. Our aim was to investigate whether MPV is a predictor of variceal bleeding in patients with cirrhotic portal hypertension.

## MATERIALS AND METHODS

### Study Design

The study was performed in accordance with the principles of the Helsinki Declaration and approved by the local Institutional Review Board. Written informed consent was obtained from all subjects, a legal surrogate, the parents, or legal guardians. A total of 62 patients with liver cirrhosis who were followed up and treated in the internal diseases department of our institution were enrolled in the study. Two groups were constituted with respect to presence of GIS bleeding. Group I comprised 31 cirrhotic patients without GIS bleeding, whereas group II was made up of 31 cirrhosis patients with GIS bleeding.

The patients were questioned for the etiology of portal hypertension, duration of the disease, beta-blocker, and nonsteroidal anti-inflammatory drug (NSAID) use. Complete blood counts, serum liver enzymes [aspar-tate transferase (AST), alanine transferase (ALT)], renal function tests (urea, creatinine), albumin, prothrombin time (PT), and total bilirubin values were analyzed. On abdominal ultrasonography, the presence of ascites and dimensions of the spleen was sought. On physical examination, the presence of encephalopathy was evaluated. Ascites and encephalopathy were graded according to Child-Pugh-Turcotte classification. Endoscopic findings and presence of varices were evaluated by gastroenterologists. Esophageal varices were staged between F1 and F4.

### Statistical Analysis

Data were analyzed using the IBM Statistical Package for Social Sciences version 10 (Inc., Chicago, Illinois, USA). Parametric test (independent samples t-test) was applied to data of normal distribution and nonparametric test (Mann-Whitney U-test) was used for the analysis of data with questionably normal distribution. The distribution of categorical variables in both groups was compared using Pearson chi-square test. A univariate analysis of potential factors on bleeding was performed with the log-rank test for categorical factors and with the univariate Cox analysis for continuous variables. Continuous data were presented as mean ± standard deviation or median (minimum-maximum), as appropriate. All differences associated with a chance probability of 0.05 or less were considered as statistically significant.

## RESULTS

A total of 31 patients (21 males, 10 females) without GIS bleeding (group I) and 31 patients (23 males, 8 females) with GIS bleeding (group II) were included in the study. A significant difference was not observed regarding the effects of age, duration of the disease, and gender of the patient on bleeding (p > 0.05) (Table 1). In group I, encephalopathy was not detected in 45.2% of the patients, while prolongation of PT was noted in 54.8%, tense ascites in 61.3%, Child-Pugh-Turcotte stage C in 67.7% of the patients respectively. However, in group II, encephalopathy was not observed in 41% of the patients, while 4 to 6 second longer PT was detected in 41.9% of the patients. Tense ascites and Child-Pugh-Turcotte stage C were detected in 77.4 and 74.2% of the patients respectively. Beta-blocker was used by 74.2 and 61.3% of the patients in groups I and II respectively. Still, NSAID was used by 22.6 and 41.9% of the patients in groups I and II respectively. At the same time, a significant intergroup difference was not detected in the incidence of bleeding (p > 0.05) (Table 2). In etiological evaluation of all patients included in the study, the role of postnecrotic cirrhosis was detected in 40.3% of the patients. However, the etiology of bleeding did not differ significantly between groups (p > 0.05) (Table 3). Moreover, MPV had no significant effect on bleeding (p > 0.05).

**Table 1:** **Table 1** Characteristics of the arouDs with and without bleedina

				*Group I*		*Group II*	
Gender		Male		21		23	
		Female		10		8	
Average age (years)				60.06		53.56	
Duration of disease (years)				3.80		3.69	

**Table 2:** **Table 2** Distribution of beta-blocker and NSAID use

				*Beta-blocker use*						*NSAID use*					
Groups				No		Yes		Total		No		Yes		Total	
I		No. of patients		12		19		31		18		13		31	
		Ratio of the group (%)		38.7		61.3		100		58.1		41.9		100	
		Ratio of drug users (%)		60		45.2		50		42.9		65		50	
II		No. of patients		8		23		31		24		7		31	
		Ratio of the group (%)		25.8		74.2		100		77.4		22.6		100	
		Ratio of drug users (%)		40		54.8		50		57.1		35		50	
Total		No. of patients		20		42		62		42		20		62	
		Ratio of the group (%)		32.3		67.7		100		67.7		32.3		100	
		Ratio of drug users (%)		100		100		100		100		100		100	

**Table 3:** **Table 3** Distribution of patients based on etiologies of portal hypertension

Groups				*HBV*		*HCV*		*Cryptogenic*		*Alcoholic*		*Wilson*		*Budd-Chiari*		*Sclerosing cholangitis*		*Alcohol* *+ HBV*		*Alcohol* *+ HCV*		*Total*	
I		No. of patients		12		2		10		3		1		1		0		1		1		31	
		Ratio of the group (%)		38.7		6.5		32.3		9.7		3.2		3.2		0		3.2		3.2		100	
		Ratio of PHT etiology (%)		63.2		33.3		43.5		33.3		100		100		0		100		100		50	
II		No. of patients		7		4		13		6		0		0		1		0		0		31	
		Ratio of the group (%)		22.6		12.9		41.9		19.4		0		0		3.2		0		0		100	
		Ratio of PHT etiology (%)		36.8		66.7		56.5		66.7		0		0		100		0		0		50	
Total		No. of patients		19		6		23		9		1		1		1		1		1		62	
		Ratio of the group (%)		30.6		9.7		37.1		14.5		1.6		1.6		1.6		1.6		1.6		100	
		Ratio of PHT etiology (%)		100		100		100		100		100		100		100		100		100		100	

HBV: Hepatitis B virus; HCV: Hepatitis C virus; PHT: Portal hypertension

**Table 4:** **Table 4** Mean variables of the cirrhotic patients with and without variceal bleeding

*Variable*		*Group II*		*Group I*		*p-value*	
AST (U/L)		72.29		66.83		0.79	
ALT (U/L)		38.06		33.25		0.51	
Urea (mg/dL)		65.77		51.83		0.31	
Creatinine (mg/dL)		1.27		0.92		0.09	
WBC (cells/μL)		7.34		6.74		0.62	
RBC (cells/μL)		3.22		3.38		0.41	
Hb (g/dL)		9.09		10.32		0.02*	
Hct (%)		25.74		29.47		0.02*	
MCV (fL/cell)		80.49		86.47		0.11	
Plt (cells/μL)		101.74		113		0.47	
MPV (fL)		7.9		8.6		0.46	
Spleen size on USG (mm)		137.16		137.8		0.95	
Bilirubin (mg/dL)		3.35		3.48		0.89	
Albumin (g/dL)		2.68		2.62		0.73	
Child score		10.54		10.25		0.85	

*Statistically significant; WBC: White blood cell count; RBC: Red blood cell count; Hb: Hemoglobin; Hct: Hematocrit; MCV: Mean corpuscular volume; USG: Ultrasonography

In 29% of the patients in group I, any endoscopic finding was not found. Histopathological results of group I patients were as follows: Antral gastritis, (32.3%), erosive gastritis (61.1%), isolated esophageal varices (77.4%), stage 2 varices (41.9%), while in 22.6% of the patients’ varices were not seen. In group II, any endoscopic sign of varicose veins was not detected. Histopathological report indicated antral gastritis (35.5%), isolated esophageal varices (90.3%), stage 3 varices, while in 6.5% of the patients, varices were not detected. Spearman correlation test evaluated correlations between groups and variables for presence and grade of varices effective on bleeding varices, which were found to be significant (p < 0.005) (Table 4). Risk of bleeding increased in line with higher grades of varices.

## DISCUSSION

In the present study, we attempted to detect whether MPV can be used as a noninvasive and practical predictive tool for variceal bleeding, which is a health problem with higher mortality and morbidity rates on portal hypertension. However, our results did not confirm any predictive value of MPV in these patients.

In Child-Pugh classification, thrombocytopenia, splenomegaly, and presence of ascites were detected as individual determinants of large esophageal varices.^13,14^ Goh et al^15^ found thrombocytopenia, splenomegaly, and presence of ascites as predictors of the presence of bleeding. Giannini et al^4^ detected platelet count/diameter of spleen ratio as an independent novel parameter related to the presence of varices. In parallel with increase in Child-Pugh stage, they observed grades II and III varices more frequently and detected increased risk of bleeding from esophageal varices.^16^ We also noted a correlation between the bleeding and the presence and grade of varices (p < 0.05). We observed increased risk of bleeding with higher grade of esophageal varices.

The use of nonselective beta-blocker decreases portal pressure and, because of the risk of variceal bleeding, also decreases mortality rates.^17^ As a result of meta-analysis, an average decrease of 40% in the risk of bleeding and 20% decrease in mortality have been reported with propranolol use.^18^ Still propranolol decreases the recurrence of esophageal varices as a complication of cirrhosis.^19^ In the present study, though not statistically significant, increased rate of beta-blocker use in the group with nonbleeding varices was worth mentioning. Prostaglandins play a critical role in the maintenance of the integrity and repair of gastroduo-denal mucosa. Therefore, disruption of prostaglandin synthesis may impair mucosal defense mechanism and repair by means of systemic mechanisms and facilitate mucosal injury.

The NSAIDs inhibit prostaglandin synthesis in the gastrointestinal mucosa leading to gastrointestinal injury.^20^ Risk of development of peptic ulcer in NSAID user is 5 to 10 times more frequent when compared with nonusers.^21^ In our study, though not statistically significant, in the group with bleeding varices, NSAID was more frequently used.

Cirrhosis demonstrates differences in socioeconomic and cultural factors. Alcohol use is the predominant etiology in Western Europe and Northern America. In the Far East, Middle East, and in our country, which is in this zone, viral hepatitis is the most important factor. As etiological factors for cirrhosis, viral hepatitis and alcoholic hepatitis were detected in 60 and 11% of the cases respectively, while any etiology was not found in 16% of the patients.^22^

In the current literature, etiology of cirrhosis has been investigated on large series. Based on the analysis of data derived from 33,379 patients, Michitaka et al^23^ suggested that hepatitis B virus 13.9%, hepatitis C virus 60.9%, alcohol 13.6%, primary biliary cirrhosis 2.4%, and autoimmune hepatitis 1.9% were responsible for the development of cirrhosis. Mendez-Sanchez et al^24^ reported that the etiology of liver cirrhosis was alcohol in 587 (39.5%), hepatitis C virus in 544 (36.6%), cryptogenic in 154 (10.4%), primary biliary cirrhosis in 84 (5.7%), hepatitis B virus in 75 (5.0%), and other in 42 (2.8%) patients.

In our study group, etiologic factors in our patients were compliant with those of our country. We attributed an excessive number of cryptogenic cirrhosis patients in our study group to scarce number of patients, retrospective design of the study, and lower level of primary health care services when compared with those of the developed countries.

The weaknesses of our study include small sample size and data restricted to the experience of a single institution. Furthermore, impacts of social, ethnic, environmental, and genetic factors on our outcomes cannot be ignored. Although some studies have found that MPV is associated with different types of bleed-ing,^6,12^ in our study we could not determine the positive correlation between platelet size and variceal bleeding.

Platelets take substantial part in primary hemo-stasis, and their size and morphology are associated with its hemostatic function.^25^ This study is the first publication to evaluate the role of MPV as an easily accessible potential marker in variceal bleeding due to cirrhosis.

## CONCLUSION

To conclude, we found a correlation between the grade of varices and likelihood of variceal bleeding; however, any relationship between MPV and bleeding could not be established. We suggest that further studies should be carried out on the predictive roles of noninvasive tests for probability of esophageal variceal bleeding.
